# In Silico Physicochemical Characterization of Fusion Proteins from Emerging Amazonian Arboviruses

**DOI:** 10.3390/life13081687

**Published:** 2023-08-04

**Authors:** Crislaine S. Leal, Carlos Alberto M. Carvalho

**Affiliations:** Graduate Program in Parasite Biology in the Amazon, Center for Biological and Health Sciences, University of Pará State, Belém 66095-662, PA, Brazil; crislaineseabra@hotmail.com

**Keywords:** arboviruses, computational biology, emerging communicable diseases, physicochemistry, viral fusion proteins

## Abstract

Mayaro (MAYV), Saint Louis encephalitis (SLEV), and Oropouche (OROV) viruses are neglected members of the three main families of arboviruses with medical relevance that circulate in the Amazon region as etiological agents of outbreaks of febrile illnesses in humans. As enveloped viruses, MAYV, SLEV, and OROV largely depend on their class II fusion proteins (E1, E, and Gc, respectively) for entry into the host cell. Since many aspects of the structural biology of such proteins remain unclear, the present study aimed at physicochemically characterizing them by an in silico approach. The complete amino acid sequences of MAYV E1, SLEV E, and OROV Gc proteins derived by conceptual translation from annotated coding regions in the reference sequence genome of the respective viruses were obtained from the NCBI Protein database in the FASTA format and then submitted to the ClustalO, Protcalc, Pepstats, Predator, Proscan, PCprof, Phyre2, and 3Drefine web servers for the determination of sequence identities, the estimation of residual properties, the prediction of secondary structures, the identification of potential post-translational modifications, the recognition of antigenic propensities, and the modeling/refinement of three-dimensional structures. Sequence identities were 20.44%, 18.82%, and 13.70% between MAYV/SLEV, SLEV/OROV, and MAYV/OROV fusion proteins, respectively. As for the residual properties, MAYV E1 and SLEV E proteins showed a predominance of the non-polar profile (56% and 55% of the residues, respectively), whereas the OROV Gc protein showed a predominance of the polar profile (52% of the residues). Regarding predicted secondary structures, MAYV E1 and SLEV E proteins showed fewer alpha-helices (16.51% and 15.17%, respectively) than beta-sheets (21.79% and 25.15%, respectively), while the opposite was observed in the OROV Gc protein (20.39% alpha-helices and 12.14% beta-sheets). Regarding post-translational modifications, MAYV E1, SLEV E, and OROV Gc proteins showed greater relative potential for protein kinase C phosphorylation, *N*-myristoylation, and casein kinase II phosphorylation, respectively. Finally, antigenic propensities were higher in the *N*-terminus half than in the *C*-terminus half of these three proteins, whose three-dimensional structures revealed three distinctive domains. In conclusion, MAYV E1 and SLEV E proteins were found to share more physicochemical characteristics with each other than the OROV Gc protein, although they are all grouped under the same class of viral fusion proteins.

## 1. Introduction

Arboviruses constitute a diverse group of viral families and include emerging pathogens transmitted from infected to susceptible hosts by a wide variety of arthropod vectors, such as biting mosquitoes, ticks, and flies [1]. Of the known species, hundreds are responsible for causing zoonotic diseases, maintained in transmission cycles between vectors and vertebrate reservoirs [2]. Among the circulating arboviruses, those belonging to the families *Togaviridae*, *Flaviviridae*, and *Peribunyaviridae* account for the highest rates of human infections and currently represent a major public health threat worldwide, especially in the tropical zone, where high temperature and humidity favor the proliferation of arthropod vectors and, therefore, the transmission of these viruses [3].

As enveloped particles, arboviruses crucially rely on membrane fusion to gain access to the host cell cytosol: a complex reaction mediated by proteins located in the viral envelope that join this lipid bilayer to a host cell membrane (e.g., plasma membrane or endosomal membrane) [4]. In general, the so-called viral fusion proteins undergo conformational changes, which are triggered by specific molecular cues, such as binding to cell receptors and/or the variation in hydrogenionic potential (pH), which can lead to exposure of hydrophobic fusion loops that interact with target membranes, causing lipid mixing [5]. These proteins are grouped into three classes (I, II, and III) based on their main structural characteristics [6].

Mayaro (MAYV), Saint Louis encephalitis (SLEV), and Oropouche (OROV) viruses are neglected members of the three main families (i.e., *Togaviridae*, *Flaviviridae*, and *Peribunyaviridae*, respectively) of emerging medically relevant arboviruses in the Amazon region, where they are related to outbreaks of febrile illness in humans [7]. Previous studies have shown that MAYV, SLEV, and OROV use class II fusion proteins—called E1, E, and Gc, respectively—for entry into their host cells, but many aspects of the structural biology of such proteins remain unclear [8]. In this sense, the present research aimed to explore the primary structures of the fusion proteins of the aforementioned arboviruses by using bioinformatics tools to determine their sequence identities, estimate their residual properties, predict their secondary structures, identify their potential post-translational modifications, recognize their antigenic propensities, and model/refine their three-dimensional structures.

## 2. Materials and Methods

### 2.1. Collection of Amino Acid Sequences

The primary structures of MAYV E1 (NP_740694.1:1-436), SLEV E (YP_009329949.1:1-501), and OROV Gc (NP_982303.1:536-1359) proteins were obtained from the NCBI Protein database (https://www.ncbi.nlm.nih.gov/protein/, accessed on 31 July 2023) [9] in the FASTA format as complete amino acid sequences derived by conceptual translation from annotated coding regions in the reference sequence genome. As provided in Law 13,123/2015 and regulated by Decrees 8,772/2016 and 10,844/2021 of the Federative Republic of Brazil, this research was registered in the National System for the Management of Genetic Heritage and Associated Traditional Knowledge (https://sisgen.gov.br/, accessed on 31 July 2023), under the number AB8C48B.

### 2.2. Multiple Sequence Alignment

The amino acid sequences of MAYV E1, SLEV E, and OROV Gc proteins were aligned using seeded guide trees and hidden Markov model profile–profile techniques on the ClustalO web server (https://www.ebi.ac.uk/Tools/msa/clustalo/, accessed on 31 July 2023) [10], providing a 3 × 3 matrix of percent identities.

### 2.3. Estimation of Residual Properties

Isotopically averaged molecular weights, isoelectric points (pIs), charges over the pH range, and side chain polarities of MAYV E1, SLEV E, and OROV Gc proteins were estimated by submitting their respective amino acid sequences to the Protcalc (https://protcalc.sourceforge.net/, accessed on 31 July 2023) and Pepstats (https://www.ebi.ac.uk/Tools/seqstats/emboss_pepstats/, accessed on 31 July 2023) web servers [11].

### 2.4. Prediction of Secondary Structures

The content of secondary structures, as well as their locations in MAYV E1, SLEV E, and OROV Gc proteins, was predicted by submitting their respective amino acid sequences to the Predator web server (https://npsa-prabi.ibcp.fr/NPSA/npsa_predator.html, accessed on 31 July 2023) [12], which was set with an output width of 70 residues and secondary structure data as DSSP format.

### 2.5. Identification of Potential Post-Translational Modifications

Potential post-translational modifications in MAYV E1, SLEV E, and OROV Gc proteins were identified based on consensus patterns by submitting their respective amino acid sequences to the Proscan web server (https://npsa-prabi.ibcp.fr/NPSA/npsa_proscan.html, accessed on 31 July 2023) [12], which was set to consider a similarity level of 100% (no mismatch).

### 2.6. Recognition of Antigenic Propensities

The antigenic propensities of MAYV E1, SLEV E, and OROV Gc proteins were recognized based on the convolution of hydrophilicity, accessibility, and flexibility (HAF) scores by submitting their respective amino acid sequences to the PCprof web server (https://npsa-prabi.ibcp.fr/NPSA/npsa_pcprof.html, accessed on 31 July 2023) [12], which was set for a window size of 7 residues.

### 2.7. Modeling and Refinement of Three-Dimensional Structures

Three-dimensional structures of MAYV E1, SLEV E, and OROV Gc proteins were modeled by submitting their respective amino acid sequences to the Phyre2 web server (http://www.sbg.bio.ic.ac.uk/phyre2/, accessed on 31 July 2023) [13] in the intensive mode (i.e., complete modeling of the entire protein using multiple templates and ab initio techniques). The refinement of output models in the PDB format was carried out by the optimization of the hydrogen bonding network and atomic-level energy minimization on the 3Drefine web server (http://sysbio.rnet.missouri.edu/3Drefine/, accessed on 31 July 2023) [14], performing post-refinement model analysis with RWplus—a pairwise distance-dependent, atomic statistical potential function combined with side-chain packing orientation specificity [15].

## 3. Results

### 3.1. Sequence Identity between MAYV, SLEV, and OROV Fusion Proteins

**Figure 1:**
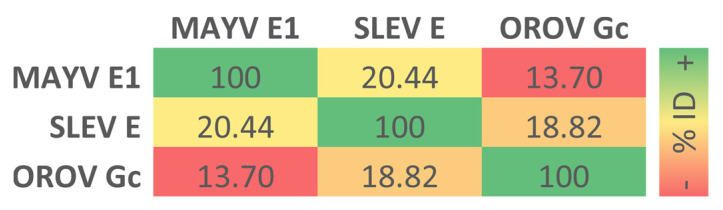
Sequence identity between MAYV, SLEV, and OROV fusion proteins. On the ClustalO web server, a multiple sequence alignment between the query proteins was generated to provide a percent of the identity matrix of their comprising residues, expressed through a red-yellow-green color gradient from minimum to maximum values.

An alignment between the amino acid sequences of MAYV E1 (436 residues), SLEV E (501 residues), and OROV Gc (824 residues) proteins revealed that sequence identity was highest for MAYV/SLEV fusion proteins (20.44%) and lowest for MAYV/OROV fusion proteins (13.70%), with SLEV/OROV fusion proteins showing an intermediate value for this variable (18.82%) (Figure 1). Considering these three proteins together, 19 positions of their amino acid sequences had a single, fully conserved residue, 30 positions had a conservation between groups of strongly similar properties, and 37 positions had a conservation between groups of weakly similar properties (Figure S1).

### 3.2. Residual Properties of MAYV, SLEV, and OROV Fusion Proteins

**Figure 2:**
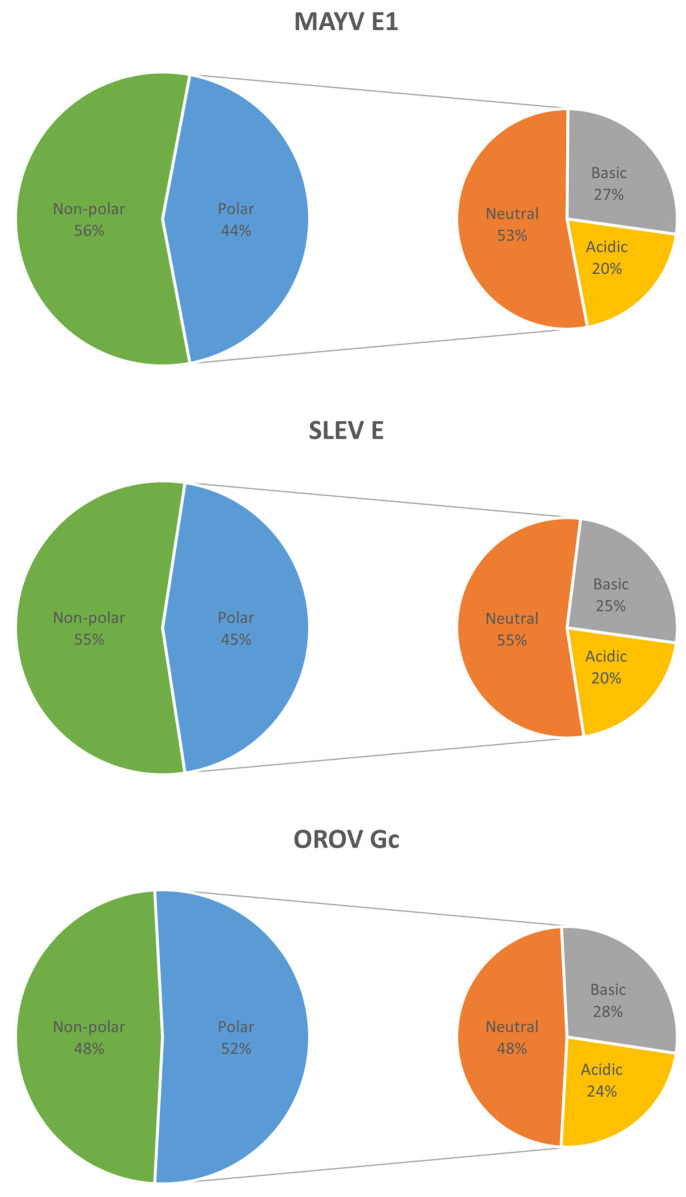
Residual properties of MAYV, SLEV, and OROV fusion proteins. On the Pepstats web server, residues of the query proteins were grouped according to polarity as non-polar (A + C + F + G + I + L + M + P + V + W + Y) or polar (D + E + H + K + N + Q + R + S + T), the latter being further grouped as neutral (N + Q + S + T), basic (H + K + R), or acidic (D + E) according to charge.

The molecular weights of MAYV E1, SLEV E, and OROV Gc proteins were 47.47, 54.05, and 93.71 kDa, while their pIs were 7.41, 7.42, and 6.99, respectively. The charges at pH 7.4 were 0.1, 0.1, and −5.5 for these three proteins, respectively; however, over pHs from 6.5 to 5.5, protein charges increased to 7.1–15.5, 5.7–13.4, and 7.8–25.0, respectively. MAYV E1 and SLEV E proteins showed a predominance of the non-polar profile (56% and 55% of the residues, respectively), whereas OROV Gc protein showed a predominance of the polar profile (52% of the residues); among polar residues, there was a preponderance of neutral over charged (basic plus acidic) residues in the first two proteins (53–55% vs. 45–47%, respectively) and the opposite was seen in the last one (48% vs. 52%, respectively) (Figure 2).

### 3.3. Secondary Structures in MAYV, SLEV, and OROV Fusion Proteins

**Figure 3:**
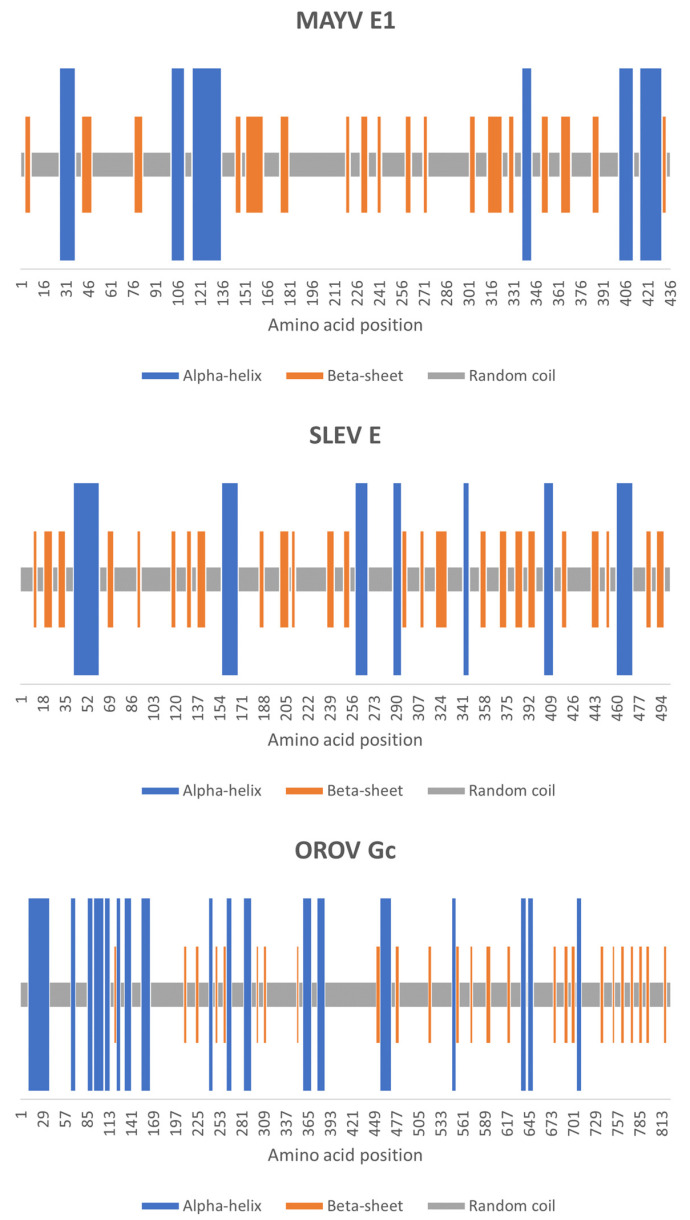
Secondary structures in MAYV, SLEV, and OROV fusion proteins. On the Predator web server, residues of the query proteins were assigned based on long-range hydrogen bonding patterns to alpha-helices, beta-sheets, or random coils, whose locations were identified in the polypeptide chains.

Although random coils prevailed in the predicted content of secondary structures of MAYV E1, SLEV E, and OROV Gc proteins (61.70%, 59.68%, and 67.48%, respectively), the first two proteins showed fewer alpha-helices (16.51% and 15.17%, respectively) than beta-sheets (21.79% and 25.15%, respectively), while the opposite was observed in the last one (20.39% alpha-helices and 12.14% beta-sheets). Furthermore, the MAYV E1 protein showed a mix of predicted alpha-helices, beta-sheets, and random coils only in the *N*-terminus and *C*-terminus thirds, as the central third was predicted to have just the last two secondary structures, while alpha-helices, beta-sheets, and random coils were predicted to span over the three-thirds of SLEV E and OROV Gc proteins (Figure 3).

### 3.4. Potential Post-Translational Modifications in MAYV, SLEV, and OROV Fusion Proteins

**Figure 4:**
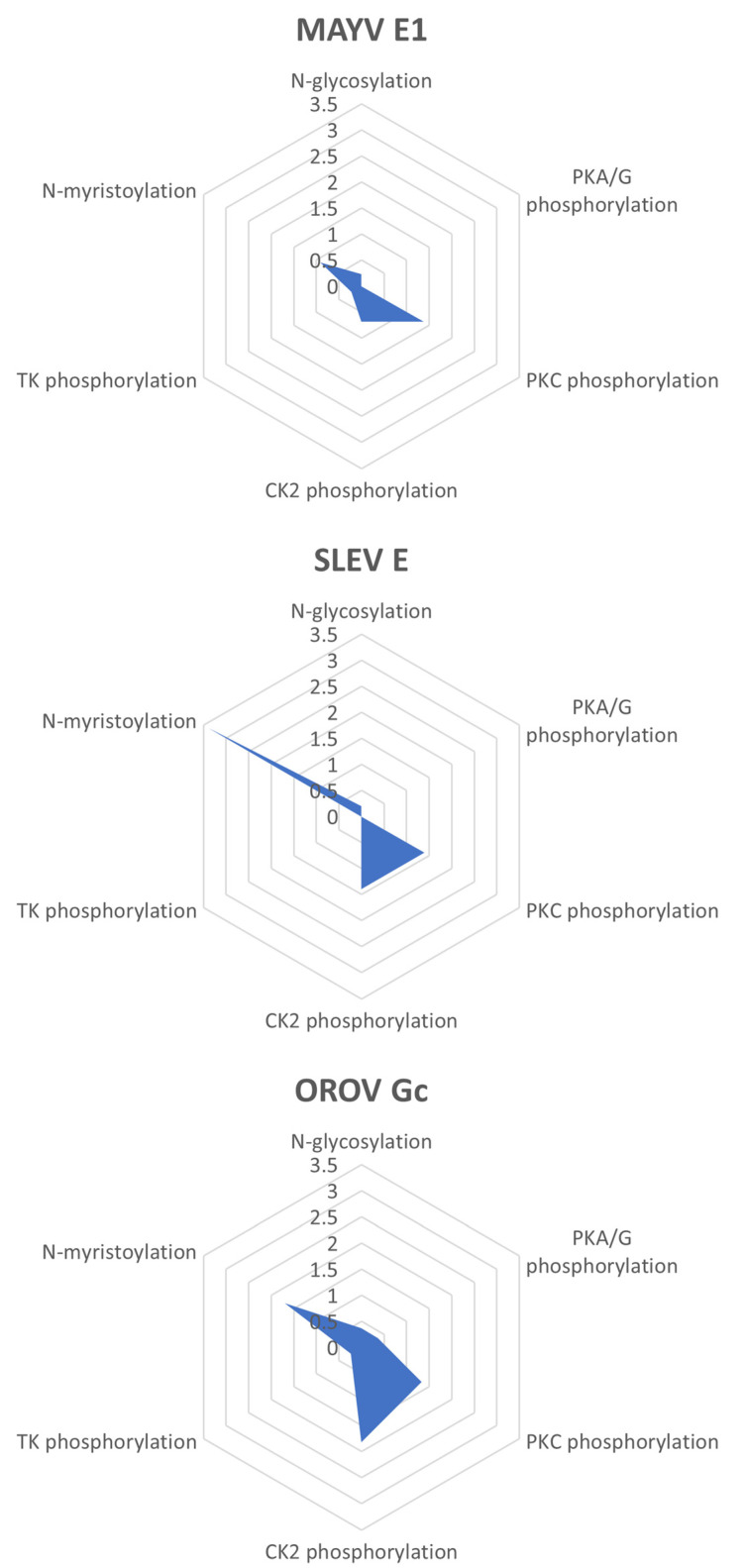
Potential post-translational modifications in MAYV, SLEV, and OROV fusion proteins. On the Proscan web server, amino acid sequences of the query proteins were scanned for *N*-glycosylation (N–{P}–[ST]–{P}, where N is the glycosylation site), PKA/G phosphorylation ([RK](2)–x–[ST], where S or T is the phosphorylation site), PKC phosphorylation ([ST]–x–[RK], where S or T is the phosphorylation site), CK2 phosphorylation ([ST]–x(2)–[DE], where S or T is the phosphorylation site), TK phosphorylation ([RK]–x(2,3)–[DE]–x(2,3)–Y, where Y is the phosphorylation site), and *N*-myristoylation (G–{EDRKHPFYW}–x(2)–[STAGCN]–{P}, where G is the *N*-myristoylation site) consensus patterns, whose occurrences per 100 residues were plotted.

Consensus patterns for *N*-glycosylation, protein kinase C (PKC) phosphorylation, casein kinase II (CK2) phosphorylation, and *N*-myristoylation were found in the three query proteins, but to different extents: MAYV E1, SLEV E, and OROV Gc proteins showed greater relative potential for PKC phosphorylation (1.38 sites per 100 residues), *N*-myristoylation (3.39 sites per 100 residues), and CK2 phosphorylation (1.82 sites per 100 residues), respectively. Regarding *N*-glycosylation, MAYV E1 and SLEV E proteins each had a single site (at amino acid positions 141 and 154, respectively), and the OROV Gc protein had three sites (at amino acid positions 79, 141, and 622). Moreover, consensus patterns for a tyrosine kinase (TK) phosphorylation were only found in MAYV E1 and OROV Gc proteins (0.23 and 0.24 sites per 100 residues, respectively), while consensus patterns for cAMP- and cGMP-dependent protein kinase (PKA/G) phosphorylation were only found in the latter (0.36 sites per 100 residues) (Figure 4).

### 3.5. Antigenic Propensities of MAYV, SLEV, and OROV Fusion Proteins

**Figure 5:**
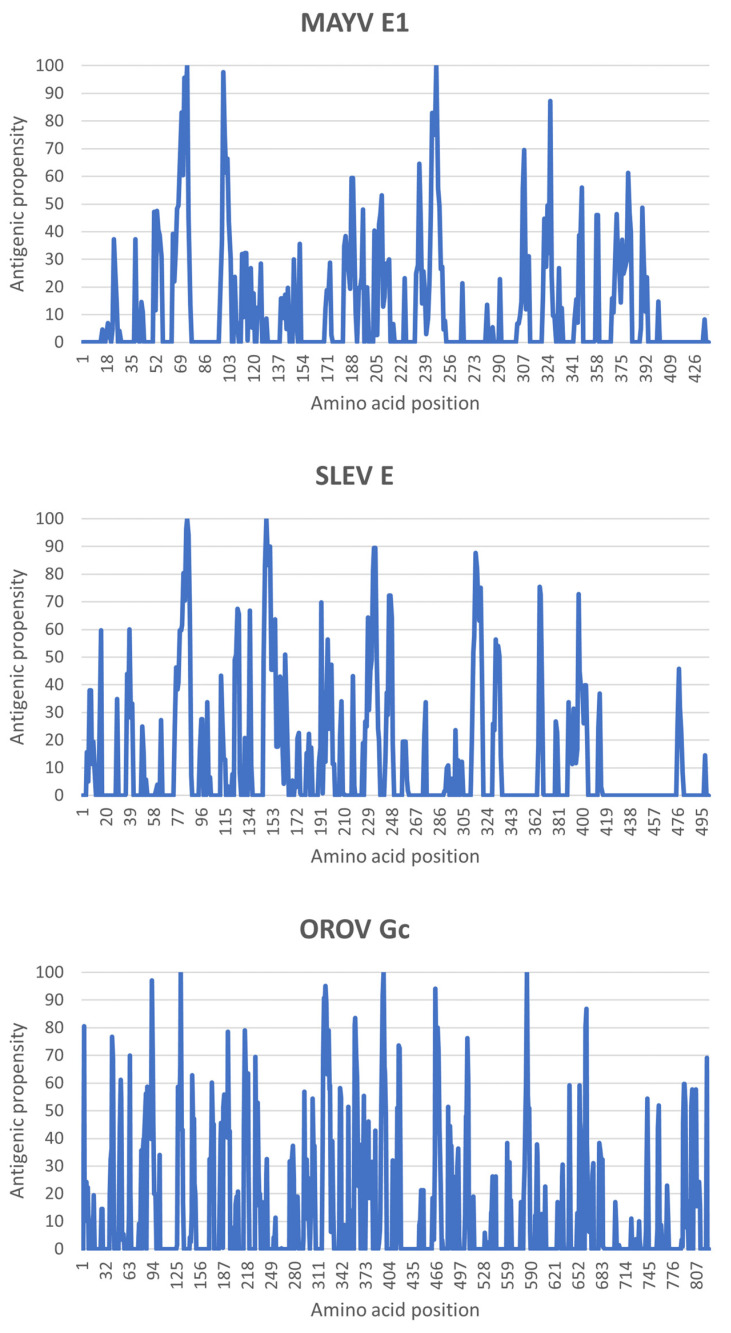
Antigenic propensities of MAYV, SLEV, and OROV fusion proteins. On the PCprof web server, amino acid sequences of the query proteins were analyzed for HAF scores of their residues, which were convoluted to provide respective antigenic propensities along the polypeptide chains.

The convolution of HAF scores resulted in higher antigenic propensities in the *N*-terminus half than in the *C*-terminus half of the three query proteins. Specifically, such propensities reached their maximum around residues Pro74/Arg247 for the MAYV E1 protein, Arg85/Asp148 for the SLEV E protein, and Asp131/Pro397/Gln585 for the OROV Gc protein (Figure 5).

### 3.6. Three-Dimensional Structures of MAYV, SLEV, and OROV Fusion Proteins

**Figure 6:**
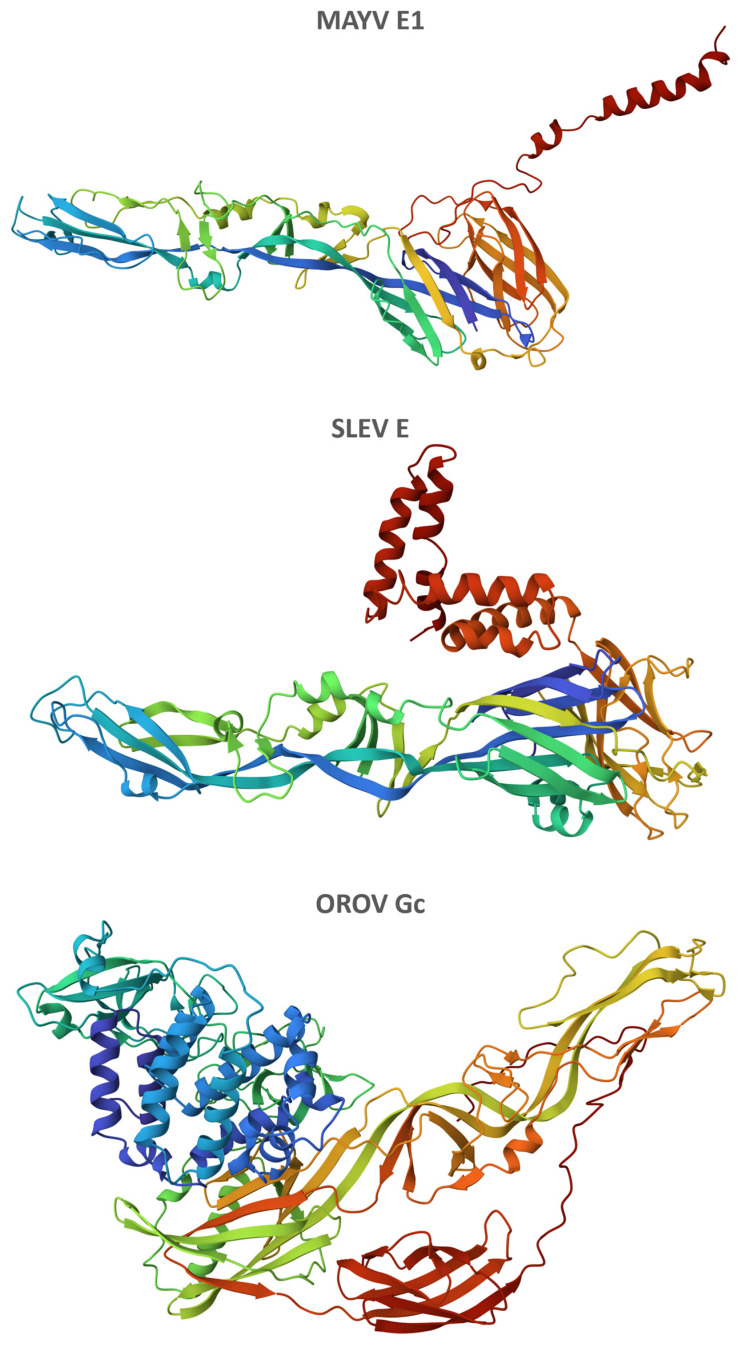
Three-dimensional structures of MAYV, SLEV, and OROV fusion proteins. On the Phyre2 web server, at least 97% of residues were modeled at >90% confidence based on single or multiple templates from the RCSB Protein Data Bank (https://www.rcsb.org/, accessed on 31 July 2023): 7KO8 for MAYV E1 protein, with 3 residues modeled ab initio; 5WSN for SLEV E protein, with 1 residue modeled ab initio; and 6H3X/7A57/6H3S/7A56 for OROV Gc protein, with 21 residues modeled ab initio. Models refined on the 3Drefine web server presenting the lowest RWplus scores were selected and had residues colored based on their respective positions in the polypeptide chain (rainbow from *N*-terminus to *C*-terminus).

The best-quality refined models of three-dimensional structures of MAYV E1, SLEV E, and OROV Gc proteins obtained the following RWplus scores: −74,064.92, −91,226.75, and −164,153.76, respectively (Figure 6). Similarly, these three proteins showed three distinctive domains: two in close proximity, which were formed mainly by beta-sheets, and a more detached one, enriched in alpha-helices (Videos S1, S2, and S3).

## 4. Discussion

Despite having distinct molecular weights, MAYV, SLEV, and OROV fusion proteins shared similar pIs. Charges at a physiological pH as well as pHs typically found within early and late endosomes—endocytic compartments usually explored by arboviruses during entry into host cells—were equivalent for the first two proteins but discrepant for the last one. As transmembrane proteins evolutionarily selected for interaction with a target lipid bilayer [16], each of them showed an expected substantial content of non-polar residues, albeit in varying proportions. Polarity seemed to be a function of protein size, probably influenced by ectodomain extension, as it was lowest in the MAYV E1 protein and highest in the OROV Gc protein.

One of the distinctive features that define class II fusion proteins is the presence of beta-sheets as the major secondary structure beyond random coils [17]. Although such a characteristic was indeed observed in MAYV E1 and SLEV E proteins, OROV Gc intriguingly showed almost twice as many alpha-helices as beta sheets in addition to showing the highest content of random coils among the query proteins, suggesting the existence of an alternative structural organization for this class of viral fusion proteins.

MAYV, SLEV, and OROV fusion proteins shared four consensus patterns for post-translational modifications, including additions of carbohydrates, lipids, and small chemical groups such as phosphate, which can be critical to their functions during infection. Apart from aiding viral envelope protein folding and assembly, *N*-glycosylation often subverts the humoral immune response by sterically preventing neutralizing antibodies from penetrating and binding to epitopes on the underlying envelope protein; *N*-myristoylation is involved in processes like the targeting of proteins to membranes and the facilitation of protein–protein interactions during virus entry and exit; and phosphorylations such as those mediated by PKA/G, PKC, CK2, and TK enzymes generally alter the surface charge in viral proteins or act as a binding site for interactions with other proteins, playing roles in several signaling pathways in the host cell [18].

In addition to concentrating regions of high antigenic propensities, the *N*-terminus half of MAYV, SLEV, and OROV fusion proteins contained spatially equivalent amino acid positions where such a property was at its maximum (i.e., around residues Pro74, Arg85, and Asp131, respectively). However, a poor overlapping of immunogenic peptides amongst these emerging arboviruses was observed in the *C*-terminus half of their fusion proteins, which is in line with previous findings on distinct members of the *Togaviridae*, *Flaviviridae*, and *Peribunyaviridae* families [19].

The ectodomain of class II viral fusion proteins is known to consist of three distinctive domains: a beta-barrel (domain I), an elongated region mostly formed by beta-sheets bearing a tightly folded fusion loop (domain II), and an immunoglobulin constant (IgC)-like module (domain III) [8]. Although the first two domains could be readily discerned by their typical spatial configuration in the refined models of three-dimensional structures of MAYV E1, SLEV E, and OROV Gc proteins, the last one was enriched in alpha-helices instead of beta-sheets which are as common in IgC domains.

It is worth noting that viral fusion proteins fold in the endoplasmic reticulum of the host cell together with an accompanying protein (called E2, M, and Gn in MAYV, SLEV, and OROV, respectively) within a polyprotein precursor, which acts as a folding chaperone [20]. Since these accompanying proteins regulate the activity of the fusion protein to ensure that the reaction occurs at the right time and place, further studies aiming to provide their physicochemical characterization should also be conducted to clarify relevant aspects of the structural biology of emerging arboviruses.

## 5. Conclusions

MAYV E1 and SLEV E proteins share more physicochemical characteristics with each other than the OROV Gc protein, although they are all grouped under the same class of viral fusion proteins.

## Data Availability

The data presented in this study are available within the article and its Supplementary Material.

## Supplementary Materials

The following supporting information can be downloaded at: https://www.mdpi.com/article/10.3390/life13081687/s1. Figure S1: Multiple sequence alignment of MAYV, SLEV, and OROV fusion proteins; Video S1: Camera spin animation of three-dimensional structure of MAYV fusion protein; Video S2: Camera spin animation of three-dimensional structure of SLEV fusion protein; Video S3: Camera spin animation of three-dimensional structure of OROV fusion protein.
