# Supplementary figures and images for: In Silico Physicochemical Characterization of Fusion Proteins from Emerging Amazonian Arboviruses
Source: Life (Basel). 2023 Aug 4;13(8):1687. doi: 10.3390/life13081687 (PMC10455688; doi:10.3390/life13081687)

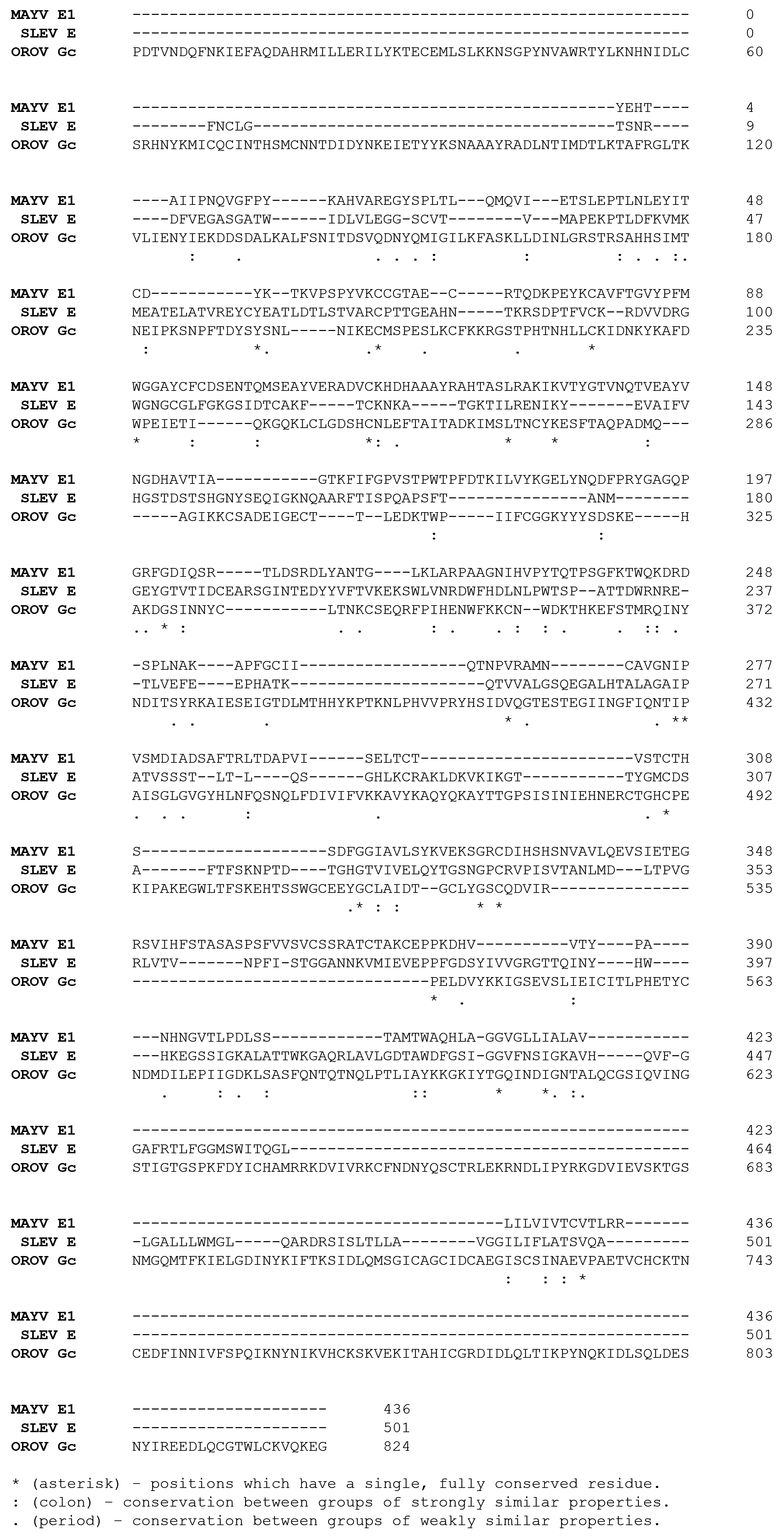

Supplement: Supplementary file 1 [file life-13-01687-s001.zip › Figure S1.png]
